# Impact of dissolved CO_2_ on calcification in two large, benthic foraminiferal species

**DOI:** 10.1371/journal.pone.0289122

**Published:** 2023-08-16

**Authors:** Linda Karoline Dämmer, Angelina Ivkić, Lennart de Nooijer, Willem Renema, Alice E. Webb, Gert-Jan Reichart

**Affiliations:** 1 Department of Ocean Systems, NIOZ Royal Netherlands Institute for Sea Research, Texel, The Netherlands; 2 Marine Biodiversity, Naturalis Biodiversity Center, Leiden, The Netherlands; 3 Department of Ecosystem & Landscape Dynamics, Institute for Biodiversity & Ecosystem Dynamics (IBED), University of Amsterdam, Amsterdam, The Netherlands; 4 Department of Earth Sciences, Faculty of Geosciences, Utrecht University, Utrecht, The Netherlands; Universita degli Studi di Urbino Carlo Bo, ITALY

## Abstract

Rising atmospheric CO_2_ shifts the marine inorganic carbonate system and decreases seawater pH, a process often abbreviated to ‘ocean acidification’. Since acidification decreases the saturation state for crystalline calcium carbonate (e.g., calcite and aragonite), rising dissolved CO_2_ levels will either increase the energy demand for calcification or reduce the total amount of CaCO_3_ precipitated. Here we report growth of two large benthic photosymbiont-bearing foraminifera, *Heterostegina depressa* and *Amphistegina lessonii*, cultured at four different ocean acidification scenarios (400, 700, 1000 and 2200 ppm atmospheric *p*CO_2_). Using the alkalinity anomaly technique, we calculated the amount of calcium carbonate precipitated during the incubation and found that both species produced the most carbonate at intermediate CO_2_ levels. The chamber addition rates for each of the conditions were also determined and matched the changes in alkalinity. These results were complemented by micro-CT scanning of selected specimens to visualize the effect of CO_2_ on growth. The increased chamber addition rates at elevated CO_2_ concentrations suggest that both foraminifera species can take advantage of the increased availability of the inorganic carbon, despite a lower saturation state. This adds to the growing number of reports showing the variable response of foraminifera to elevated CO_2_ concentrations, which is likely a consequence of differences in calcification mechanisms.

## Introduction

With globally rising atmospheric CO_2_ levels, the marine carbonate system is steadily changing, approximately 25% of the CO_2_ added to the atmosphere since the industrial revolution has been taken up in the upper layers of the ocean [1]. This uptake has decreased pH by ~0.1 units [2–5] and shifted the speciation of dissolved inorganic carbon (DIC) by decreasing the carbonate ion concentration ([CO_3_^2-^]) and increasing the bicarbonate ion concentration ([HCO_3_^-^]). As a consequence, the saturation state with respect to aragonite and calcite has been steadily declining and is widely believed to hamper marine calcification, for example by increasing the energy costs for maintaining high internal saturation states [6, 7]. However, the addition of CO_2_ to seawater also elevates the total concentration of DIC, which may be beneficial to calcification provided that the organism is capable of manipulating the ratio between the different inorganic carbon species. This may be achieved, for example, by actively increasing the pH during calcification and hence converting the (extra) HCO_3_^-^ into CO_3_^2-^, thereby increasing saturation state [8, 9]. Differences in the ability to manipulate their internal pH, may explain the observed variable responses of organisms to ocean acidification [10, 11]. Other parameters that may determine the reaction of foraminifera to ocean acidification may include increased carbon uptake by the symbionts (if present) and increased energy allocation to maintain the intracellular-extracellular ion balance [12].

Foraminifera are amongst the ocean’s most important calcifiers, with planktonic species estimated to produce up to 50% of all calcium carbonate in the open ocean [13]. In tropical regions, large benthic foraminifera can contribute up to 54% of the sediment [14–16] and approximately 80% of foraminiferal derived carbonate in reefs stems from large benthic foraminifera [16]. Since both planktonic and benthic foraminifera play a significant role in the global calcium carbonate production, it is essential to quantify and understand their response to changes in marine inorganic carbon chemistry. Since calcification produces CO_2_, the net impact of ocean acidification on calcification rates may either provide a positive or negative feedback to atmospheric CO_2_.

Among the larger benthic foraminifera the responses to ocean acidification are mixed [17], with reports showing a reduction in calcification [18–24] or standing stocks [25, 26], but also an increase in chamber addition rates or no response to experimentally-induced ocean acidification [20, 27, 28]. Whereas the response in growth rates by low Mg-foraminifera to changes in *p*CO_2_ seems to be less variable [29], the overall mixed responses of foraminiferal calcification may well indicate differences in biomineralization strategies between genetically distant groups [30]. This could be related to the presence of photosynthetic symbionts in most large benthic foraminifera and their absence in most smaller, benthic foraminifera. Although some of these species have kleptoplasts [31, 32], photosynthesis by these intact algal plastids is unlikely to have an equally large impact on calcification as algal symbionts do in the large benthic foraminifera. Photosynthesis and calcification are linked in multiple ways: both processes compete for inorganic carbon, but the local elevation of pH by the symbiont’s photosynthesis may also facilitate calcite precipitation through a higher saturation state. If the stimulus of photosynthesis is more important than the competition for inorganic carbon, the presence of symbionts may affect Mg incorporation by elevation of the precipitation rate [33, 34]. Manipulation of the internal pH is so far only recorded for species precipitating low Mg/Ca calcite, which may indicate that the strong fractionation against Mg in many rotaliid foraminifera is a direct consequence of a calcifying fluid that is well-separated from ambient seawater [35]. Species with a Mg/Ca closer to those found in inorganic precipitation experiments, may well precipitate from a more seawater-like fluid; the absence of a strong pH gradient (high inside, low outside; [36]) may reflect a regular exchange of the calcifying fluid with the surrounding seawater [37].

Here, we test the effect of elevated *p*CO_2_ on calcification in the large benthic foraminifera *Amphistegina lessonii* and *Heterostegina depressa*. These species both occur in shallow, tropical regions but differ in the chemical composition of their calcite. The former species precipitates a shell consisting of layers (lamella) with an intermediate Mg/Ca ratio (~40 mmol/mol; [38]). *H*. *depressa* also belongs to the Rotaliida, but belongs to a different superfamily of which the members precipitate calcite with a higher Mg/Ca (~150 mmol/mol; [39]). We incubate both species across a range of atmospheric *p*CO_2_ concentrations (400–2200 ppm) and determine net calcification by monitoring changes in alkalinity. Moreover, we cross-calibrate the alkalinity changes with variability in chamber addition and, for *A*. *lessonii*, chamber wall thickness measured using MicroCT-scanning. The combination of these results can be used to increase our understanding of the interactions between marine inorganic carbonate chemistry and foraminiferal calcification, and could therefore help predicting and understanding the reaction of one of the most important calcifiers, large benthic foraminifera, to future climate change.

## Material and methods

### Sample collection and culture

Specimens of the large benthic foraminifera *Amphistegina lessonii* and *Heterostegina depressa* were isolated from sediment collected at the tropical reef aquarium at Burgers’ Zoo in Arnhem, The Netherlands [40]. Living specimens were identified by their homogeneously coloured yellow/brown cytoplasm, pseudopodial activity and motility of specimens. After isolation, they were divided into groups of 50 (*A*. *lessonii*) or 25 (*H*. *depressa*) specimens of comparable sizes (approximately 500–600μm diameter for *A*. *lessonii*) and placed in flasks filled with 50 ml of filtered (5μm) North Atlantic sea water. For each of the two species, one flask per CO_2_ condition contained 5 mg/L fluorescent calcein indicator to stain newly formed carbonate. A solution of freeze-dried *Dunaliella salina* was added to all flasks, that were placed at 24°C for seven days. After this pre-staining period, calcein-marked specimens were examined under a Zeiss Axioplan 2 microscope, equipped with appropriate excitation and emission optics to identify newly formed chambers. Those individuals with one or more fluorescent chambers were placed back into the culturing flasks and the calcein-containing media were replaced with filtered, calcein-free seawater. This water was exposed to an atmosphere corresponding to that of the experimental condition to allow for pre-equilibration of the carbonate system in the water for one week.

This procedure resulted in an experiment with four groups of three replicates for *A*. *lessonii* and four groups of two replicates (or duplicates) for *H*. *depressa*. The 50 or 25 specimens within one flask are considered pseudo-replicates and variability within as well as between culture flasks will be separately discussed to test for the potential effect of pseudo-replication. For *H*. *depressa*, one of the flasks during the pre-staining phase did not contain any specimens with stained chambers and therefore counting the number of chambers added was not possible, but calcification can still be compared with the other treatments through alkalinity measurements.

### Culturing setup

**Fig 1:**
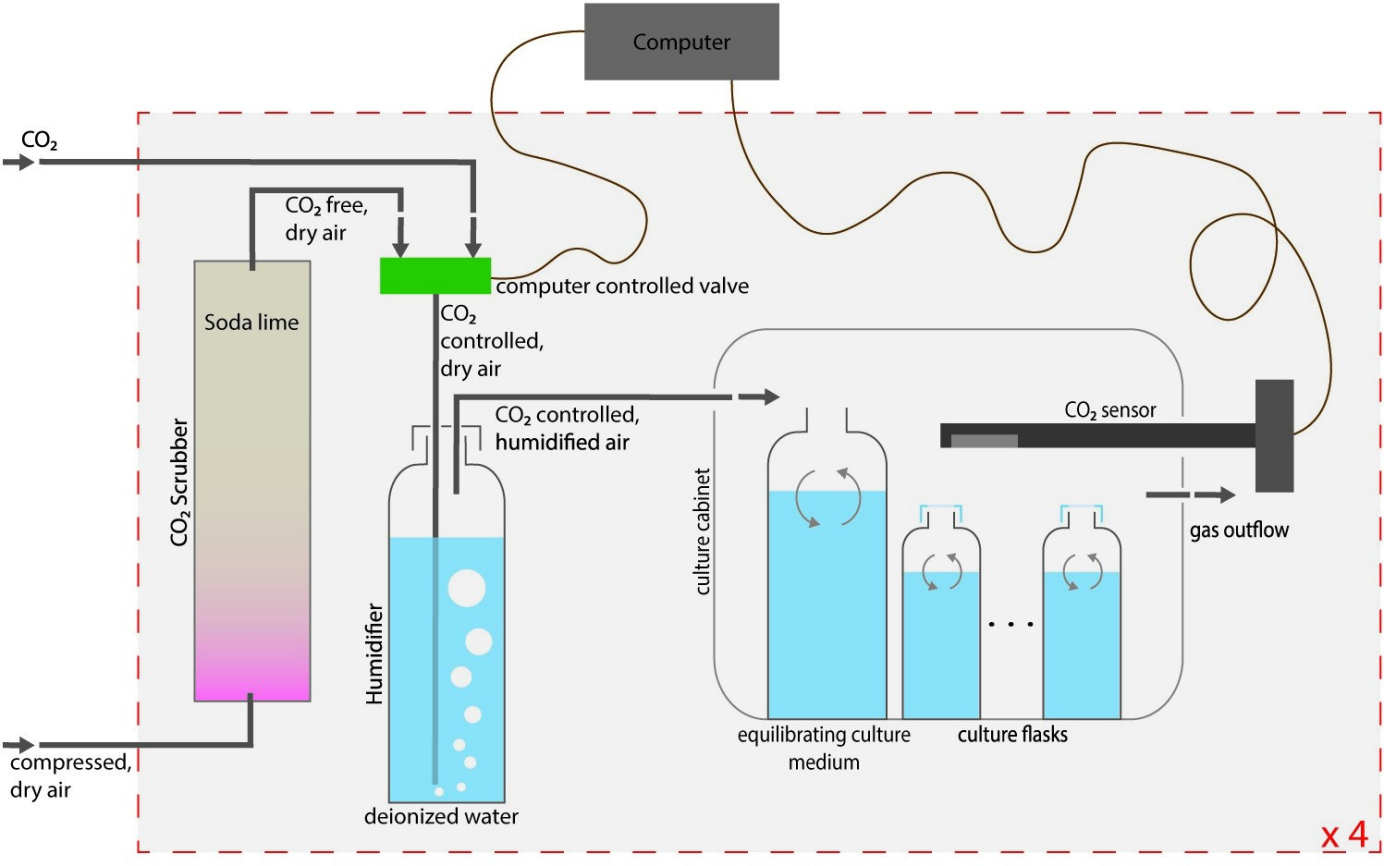
CO_2_-controlled culture set up. A control unit constantly monitored the *p*CO_2_ in four cabinets and compares them to set values. According to the offset, the ratio between pure CO_2_ and CO_2_-free air was adjusted and once humidified, added to the cabinet. This secured an atmosphere with constant *p*CO_2_ for the period of the culturing experiment. This schematic shows just one set up, everything shown within the red rectangle (CO_2_ scrubber, humidifier, and culture cabinet) was build four times and used in parallel for the experiment connected to just one computer, with a different *p*CO_2_ in each of the four chambers. The different components are not to scale.

The culture set-up (Fig 1) consisted of four chambers with controlled atmospheric composition of 18.5 L air volume. Each chamber contained a Vaisala CARBOCAP GMD20 sensor placed in the centre of the chamber, constantly monitoring atmospheric CO_2_ concentrations. Custom-made software (available from the authors upon request) based on Siemens Simatic Step 7 software automatically calculated the ratio between CO_2_-scrubbed, compressed, dried air and pure CO_2_ that needed to be added to maintain the pre-set atmospheric CO_2_ concentrations, while keeping a constant (3 Lmin^-1^) gas flow rate. The four treatments were set to CO_2_ levels of 400, 700, 1000 and 2200 ppm atm *p*CO_2_. Measured CO_2_ levels were typically within +/-10ppm for the higher concentrations and within +/-40ppm for the lower concentrations. After opening the cabinets, which was necessary to for example take water samples, CO_2_ levels returned to the set values within minutes.

The gas mixture was bubbled through a humidifying chamber filled with 1 L of de-ionized water before entering the cabinet to elevate the humidity of the gas inside the cabinet, since a very low humidity would result in high evaporation, which in turn would change salinity and other parameters. To minimize evaporation from the culturing flasks even further, flasks were closed with vented lids that allow for gas exchange. Several additional open bottles filled with 250 ml of filtered sea water were placed into each chamber to allow equilibration between the dissolved CO_2_ in these bottles’ seawater and that of the atmosphere.

These equilibrated bottles were used weekly to replace the culture media in the culturing flasks and ensured constant carbonate chemistry during the incubation period. After subsampling (see section below, ‘Carbonate chemistry of the culture media’), any remaining culture medium was discarded and replaced by fresh, pre-equilibrated culture medium. All foraminifera were then fed by adding 1 ml of a concentrated solution containing freeze-dried *D*. *salina*. This concentrated solution was prepared by suspending 32.5 mg of the algae in 100 ml of the corresponding treatment’s pre-equilibrated culture medium.

Salinity in the culture media was monitored and constant at 37.3. Temperature was kept at 23.0°C, light was switched on for 12 hours per day to simulate a natural day/night light cycle using fluorescent tubes with a light intensity of 240 μmolm^-2^s^-1^, close to that experienced in the field by both *Amphistegina* sp. and *H*. *depressa* [41], and a luminous flux of 300 lux. The transparent culture cabinets were illuminated from four sides to ensure homogeneous light conditions.

After 36 (for *H*. *depressa*) or 50 days (for *A*. *lessonii*), the experiment was terminated by rinsing the culture flasks with de-ionized water and removing the specimens with a brush. Foraminifera tests (i.e., shells) were treated following a cleaning protocol [42, 43]. Examination with fluorescent microscopy was repeated to count newly formed chambers, identified by non-stained chambers formed after those stained by calcein.

Since not all specimens used in this experiment were calcein-stained, only chamber addition of pseudo-replicates in one flask were used to calculate the total population’s growth for each treatment. The percentage of calcein-stained specimens that did not calcify during the experiment in each treatment was between 20 and 30%. Since only one of the triplicates/duplicates for each treatment was stained, to estimate the total number of calcifying specimens (including in those flasks that were not pre-stained) in each treatment, the mortality was extrapolated from the pre-stained flasks to all flasks. Between 20 and 30% of all specimens were assumed to not have formed any chambers, therefore this percentage (i.e., 25%) was subtracted from the total number of specimens. The average number of chambers formed by stained, calcifying specimens was extrapolated to the entire population of the same species and treatment (Table 2). The total number of chambers formed was calculated using the average number of chambers formed by calcifying calcein-stained specimens as representative of the entire population of the treatment.

### Carbonate chemistry of the culture media

Every week, samples were collected to measure dissolved inorganic carbon (DIC), nutrients (PO_4_, NH_4_, NO_3_, NO_2_ and SiO_2_) and for Total Alkalinity (TA) were collected from the replaced culture media. From these parameters, the entire inorganic carbon system can be calculated. Due to the small water volumes used per replicate, water samples from culturing flasks from the same incubator were combined into one aliquot to ensure sufficiently large volume for the analyses. Samples for DIC were stored in air-tight 5 mL glass vials poisoned with 15 μL mercury (II) chloride (HgCl_2_) prior to analysis.

Nutrients were analysed in a temperature-controlled laboratory equipped with a TRAACS Gas Segmented Continuous Flow Analyser. Measurements were made simultaneously on four channels for Phosphate, Ammonium, Nitrite, and Nitrate + Nitride and measured following established protocols [44–47]. All measurements were calibrated with standards diluted in low nutrient seawater with a salinity of 37 to ensure that analyses were performed within the same matrix as the samples.

DIC was measured using a Technicon TRAACS 800 autoanalyzer spectrophotometric system [48]. TA was measured using an automated spectrophotometric alkalinity system described by Liu et al. [49]. To summarize, 45 μL of bromocresol purple (10 mmol/L), which changes its colour with pH, was added to 60 ml of the sample, the mixture was then titrated with 0.1 M HCl, and the resulting changes in colour were monitored by spectrophotometry. CRM (Certified Reference Material, Dr. Dickson, Scripps Institution of Oceanography) was used as a standard material for drift correction, the measured nutrients from the same sample were used to correct the TA. Standard deviation between replicate measurements was typically ~3 μmol/kg.

The Alkalinity Anomaly Technique allows calculating calcification [50]. The precipitation of 1 mol of calcium carbonate causes a decrease of 2 moles TA [51]. The changes in TA observed between the beginning of the incubation period and at the end of the experiment is thus proportional to the amount of CaCO_3_ precipitated during that period. This result needs to be corrected for other factors, which can also alter TA in sea water, such as changes nutrient concentrations and salinity. Therefore, the following equation [52] was used to assess the amount of CaCO_3_ formed each week by each species in each treatment:

$$m(CaCO_{3})=0.5\times [\Delta TA+\Delta PO_{4}-\Delta NH_{4}+\Delta (NO_{3}+NO_{4})]\times V_{SW}\times \rho_{SW}\times M(CaCO_{3})$$

where V_SW_ is the volume of sea water used for the TA measurements in L, ρ_SW_ is the density of the sea water corrected for salinity and M(CaCO_3_) is the molar mass of CaCO_3_. The result is CaCO_3_ (in μg) precipitated by the foraminifera. The remaining carbonate system parameters (pH and saturation state Ω) were calculated with PyCO2SYS v1.6.0 [53]. The decision which of the carbonate system parameters were to be measured and which were to be calculated was based on the experimental design and technical restrictions.

### MicroCT-scanning of cultured foraminifera

A total of 19 calcein-stained *A*. *lessonii* specimens were analysed using high resolution micron-scale computed tomography (MicroCT; Zeiss Xradia 520 Versa) at Naturalis Biodiversity Center (Leiden, the Netherlands). Specimens were imaged at 80 kV using 4* optical magnification at a voxel size of 1.1–1.5 μm. and introduced into Avizo 2020.3 3D software (ThermoFisher Scientific, Waltham, MA, United States) to generate 3D models of the tests and segmentation of the chambers grown during the experiment.

Using the MicroCT-scans, a 3D-rendering of each scanned specimen was created with Fiji (ImageJ 1.53c). For each specimen, an orientation in the 3D-rendering was found, that sectioned the shell wall of the F-chamber at a 90° angle, using the pores as a guide. The correct plane was identified by pores that penetrate the entire section of the shell wall at a constant width. The correct plane was then used to measure shell wall thickness by seven randomly placed lines drawn between the outer and inner chamber wall’s surfaces. In addition, for two specimens (one grown at 700 and one grown at 2200 ppm *p*CO_2_) the volume of carbonate in the F-chamber was estimated based on the MicroCT-scans. To compare with previously published results on calcification [54], the volumes (mm^3^) were converted to weight (μg) using the density of calcite (2.71 g/cm^3^).

### Statistical treatment of the data

To test for significant differences in the average chamber addition rates and average changes in TA as a function of *p*CO_2_, a t-test was performed on combinations of two groups for each of the species (four groups in total for *A*. *lessonii* and three in case of *H*. *depressa*). The type of t-test applied assumed independent samples, since the compared data (i.e., specimens from different bottles) are not related.

An ordinary least sum of square regression analysis was performed to investigate the relationship between the number of chambers formed during the experiment and the calculated amount of carbonate produced based on the change in alkalinity. A linear regression model was assumed and significance (p-value) as well as the equation describing the regression and the residual sum of squares (R^2^) are reported. The calculated 95% confidence interval for the linear regression is also plotted.

## Results

### Inorganic carbonate system

The inorganic carbonate system of the culture media at the beginning of the culturing period (Table 1) shows that even at the highest CO_2_ concentrations (2200 ppm), the culture media remained saturated with respect to calcite (Ω>1).

**Table 1:** Seawater inorganic carbon chemistry at the onset of the experiment.

set	measured				calculated	
Temperature	Salinity	*p*CO_2_ (ppmv)	TA (μmol/kg)	DIC (μmol/kg)	pH (total scale)	Ω_Calcite_
23.0	37.3	400	2395	2080	8.05	5.22
23.0	37.3	700	2411	2210	7.85	3.55
23.0	37.3	1000	2411	2270	7.71	2.73
23.0	37.3	2200	2403	2392	7.36	1.30

Total alkalinity (TA) and dissolved inorganic carbon (DIC) concentration were measured and used to calculate pH and saturation state Ω_Calcite_.

Weekly changes in the total alkalinity varied greatly between species and CO_2_ concentrations (S1 Fig, S1 Table). For both species the changes in TA follow a similar pattern with the strongest decrease in TA occurring at 700 ppm *p*CO_2_ and the smallest decrease at the highest *p*CO_2_.

### Foraminiferal chamber addition rates

More than 70% of the *Amphistegina* specimens and almost 70% of all *Heterostegina* specimens continued adding chambers to their shell during the culturing experiment, with the highest percentage (85%) of calcifying *A*. *lessonii* specimens at 700 ppm. For *H*. *depressa*, there is no clear relation between the portion of calcifying specimens and the *p*CO_2_ (Table 2, S2 Fig).

**Table 2:** Number of chambers added per specimen during the experiment, estimated number of calcifying specimens and extrapolated number of chambers added during the experiment for both species cultured.

	Chambers added/ specimen (+/- 1SD)				Calcifying specimens		Chambers added	
*p*CO_2_ ppm	*A*. *lessonii *		*H*. *depressa *		*A*. *lessonii* (n = 150)	*H*. *depressa* (n = 50)	*A*. *lessonii *	*H*. *depressa *
400	1.94 (± 0.88)	n = 31	2.55 (± 0.88)	n = 11	108 (72%)	32 (64%)	210	82
700	2.73 (± 1.23)	n = 30	3.03 (± 1.45)	n = 15	128 (85%)	33 (66%)	349	100
1000	1.60 (± 0.73)	n = 30	NA	NA	104 (69%)	NA	166	NA
2200	1.62 (± 0.85)	n = 23	1.62 (± 0.85)	n = 12	83 (55%)	38 (76%)	134	62

Results for *H*. *depressa* incubated at 1000 ppm of CO_2_ are not available since too few of them grew chambers during pre-staining. Note the different experiment durations for both species, 50 days for *A*. *lessonii* and 36 days for *H*. *depressa*.

Similar mortality rates (20–30%) have been observed in previous culturing experiments [55] and are likely due to the transfer of specimens to other conditions (e.g., from the stock aquarium into a petri dish and then into the culture flasks). Since there is no relation between mortality and *p*CO_2_ of the experimental condition, survival is unlikely due to the change in *p*CO_2_ itself.

**Fig 2:**
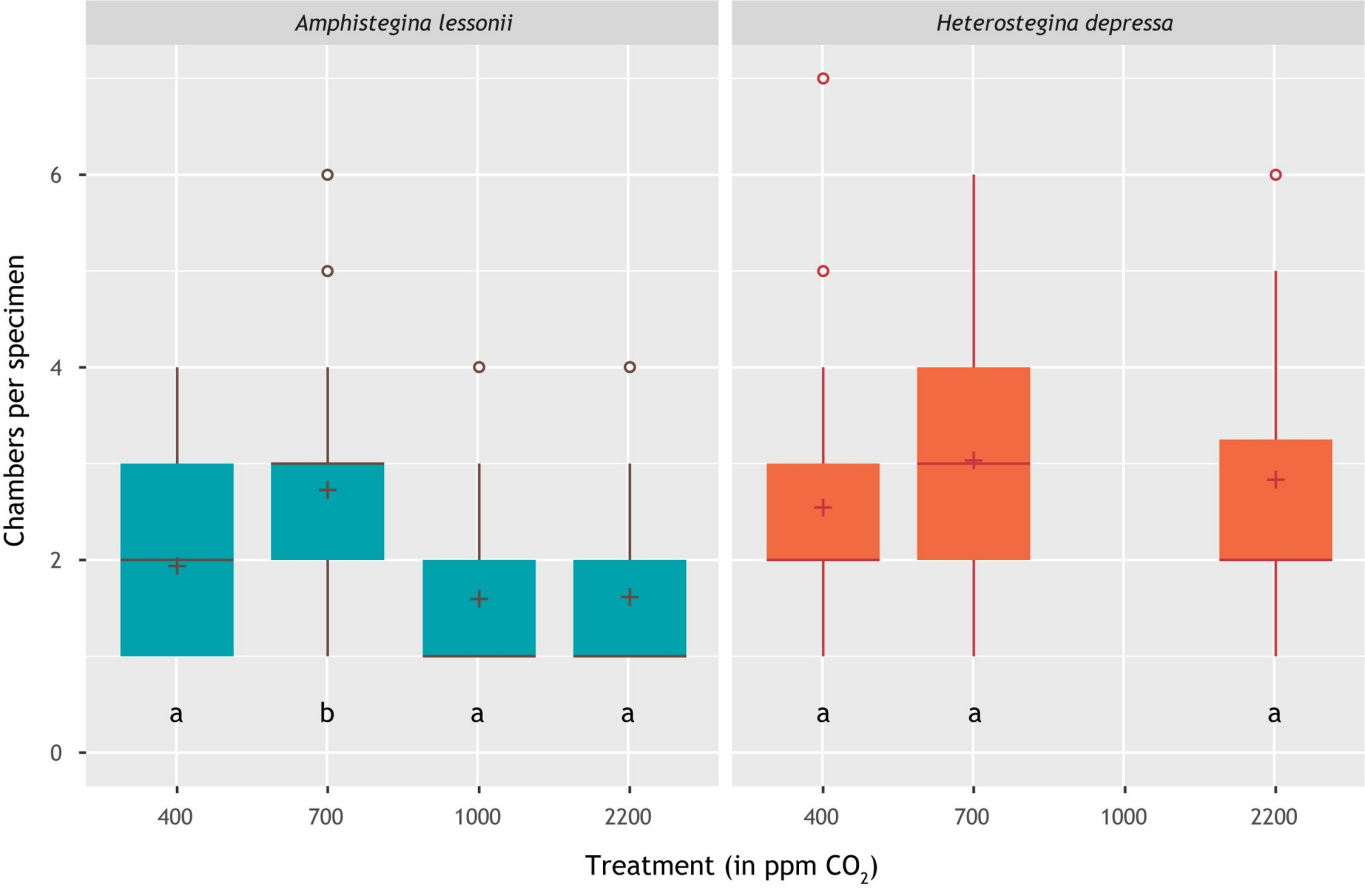
Box plot showing the number of chambers added per calcein-stained, calcifying individual (specimens with no chambers were excluded). Average values are indicated by plus symbols. Letters below the boxplots indicate statistically significantly different groups (t-test, p-value < 0.05).

For the calcifying specimens, the number of chambers that were added varied between 1 and 6 for *A*. *lessonii* and between 1 and 7 for *H*. *depressa*. Variability in chamber addition rates for the pseudo-replicates (i.e., variability between number of chambers added per specimen from one flask) was always between 45 and 55% (relative standard deviation). Combined, this shows that the total number of chambers added is more than 2.5 times higher in the 700 ppm *p*CO_2_ treatment compared to 2200 ppm *p*CO_2_ for *A*. *lessonii* and 1.6 times higher for *H*. *depressa* (Table 2; Fig 2). For both species, the extrapolated number of added chambers in the 400 ppm treatment is between the number of chambers added at 700 ppm and 2200 ppm. Chamber addition rates could not be quantified for *H*. *depressa* in the 1000 ppm treatment due to lack of calcein stained specimens. *A*. *lessonii* appears to add most chambers at the second lowest *p*CO_2_ (700 ppm) and least in the highest *p*CO_2_ (2200 ppm) treatment.

### Changes in calcification

**Fig 3:**
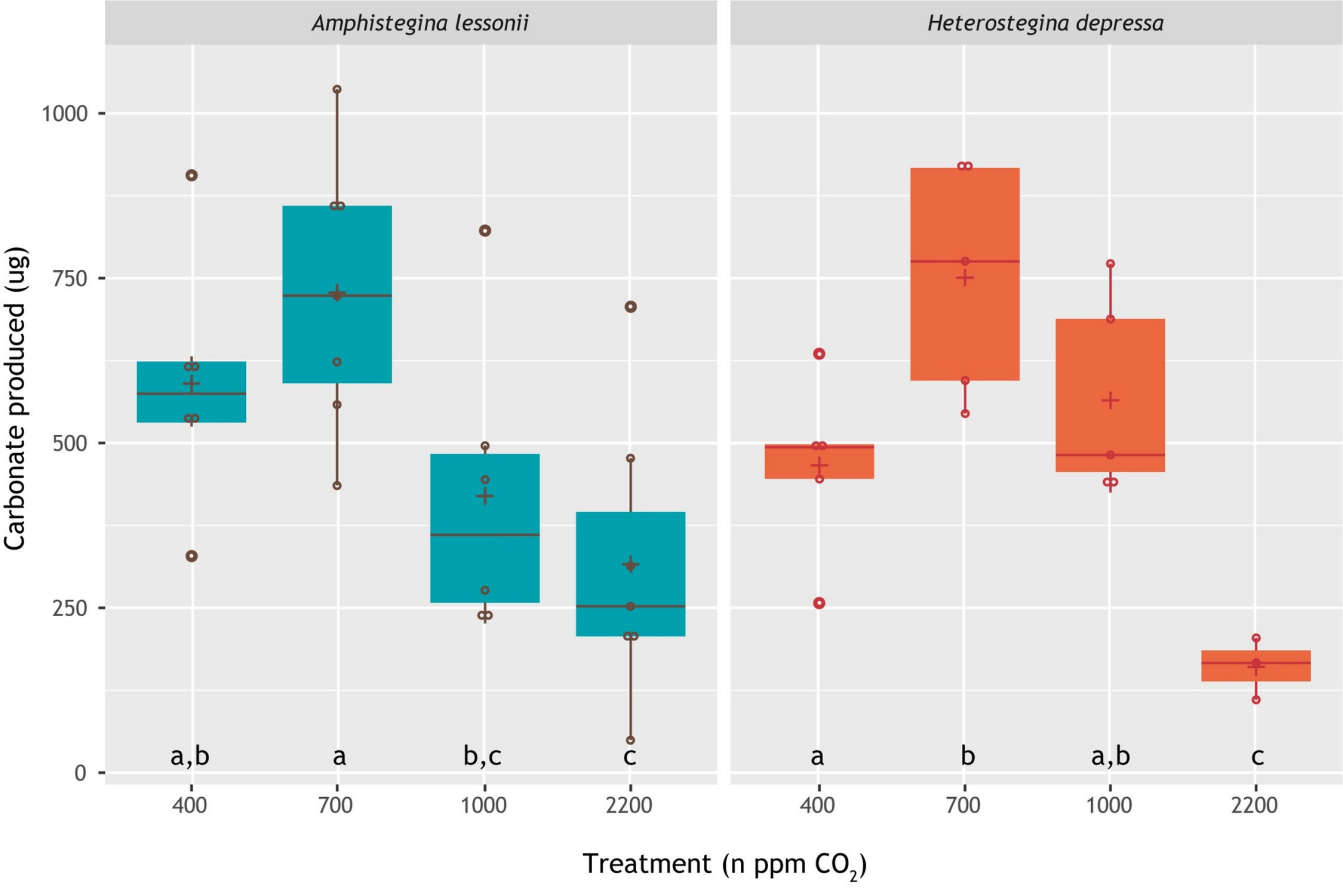
Total amount of carbonate formed in each treatment per week, calculated from weekly changes in TA. Statistically identical (t-test, p value > 0.05) treatments are indicated by the same letter. *A*. *lessonii* calcifies significantly less under higher CO_2_ concentrations (1000 and 2200 ppm) than at ambient and moderately increased levels (400 and 700 ppm). *H*. *depressa* calcifies significantly less at highest CO_2_ concentrations (2200 ppm) compared to all other treatments. Both species appear to have a calcification optimum at slightly elevated *p*CO_2_ levels.

Both species produced most new carbonate (~750 μg) at *p*CO_2_ levels of 700 ppm. Moreover, the total amount of new carbonate added was very similar between the two species (Fig 3). Incubated specimens of *H*. *depressa* produced notably less new carbonate (approximately 150 μg) at the highest *p*CO_2_ levels (2200 ppm), whereas for specimens of *A*. *lessonii*, calcification was similar at 1000 and 2200 ppm (250–350 μg).

**Fig 4:**
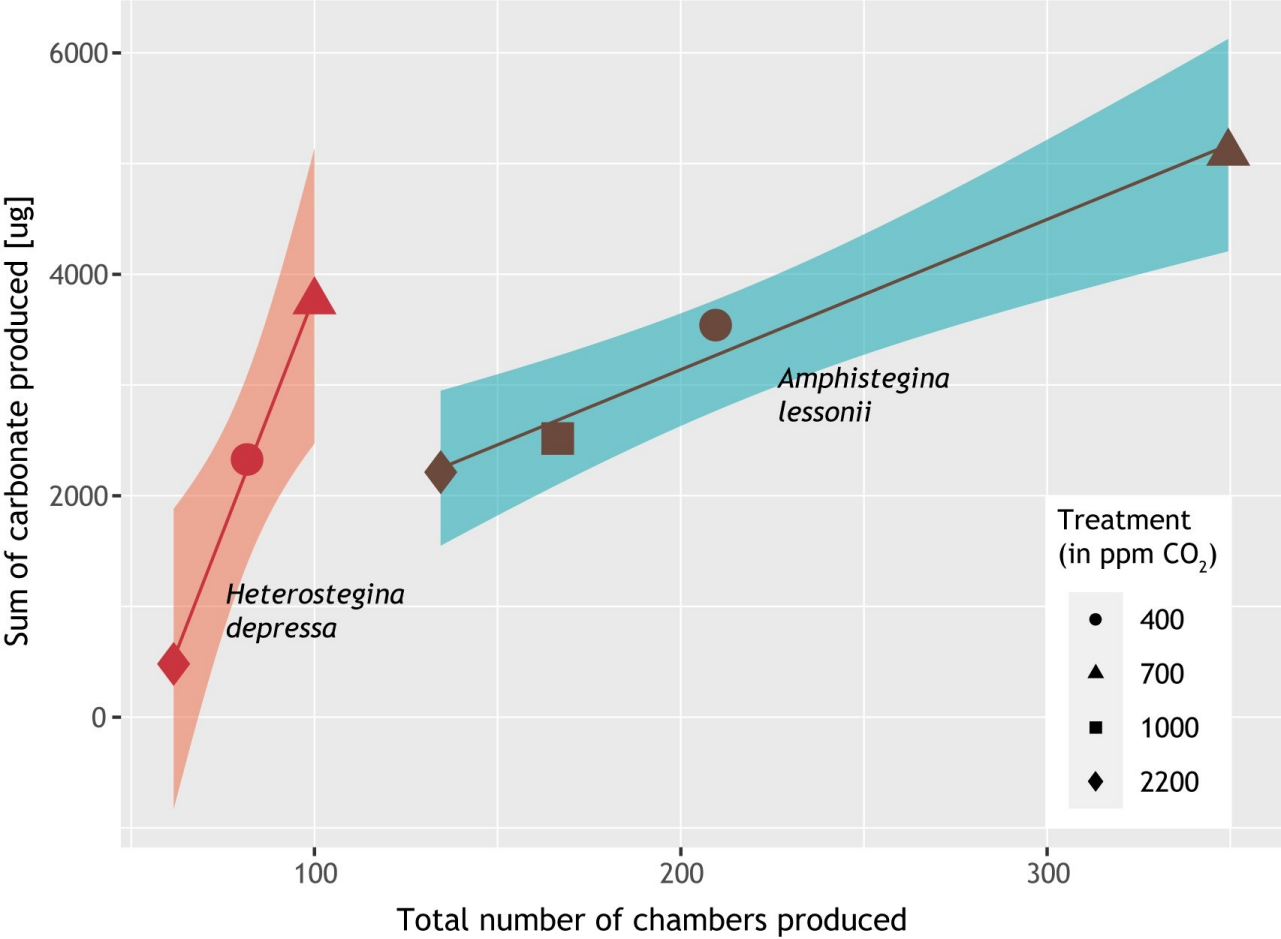
Relationship between the amount of carbonate and the number of chambers produced. The amount of carbonate formed by the foraminifera correlates linearly with the total number of chambers formed (p-values < 0.05). The relationship is described by y = 85.27(±4.26) x—4722.67(±351.65) for *H*. *depressa* (adjusted R^2^ = 0.995) and by y = 13.57(±1.43) x + 424.48(±325.22) for *A*. *lessonii* (adjusted R^2^ = 0.968). Both species produce the highest amount of carbonate and the largest number of chambers at 700 ppm *p*CO_2_ and the lowest amount of carbonate and the lowest number of chambers at 2200 ppm *p*CO_2_.

The sum of carbonate produced over the entire experiment can be compared with the number of chambers formed during the experiment (Fig 4), showing positive correlations, albeit that the slope of this dependency is steeper for *H*. *depressa* than for *A*. *lessonii*.

**Fig 5:**
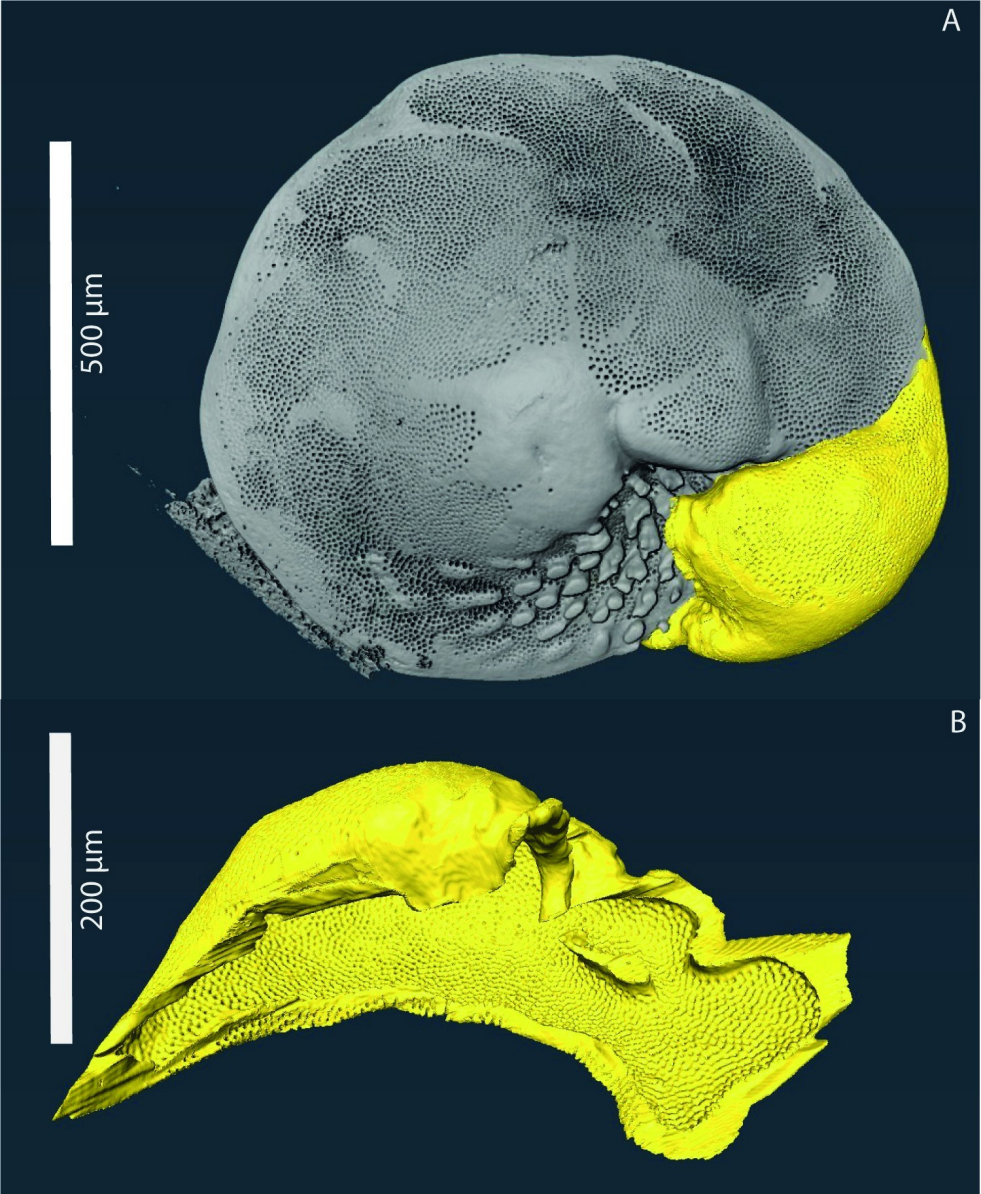
3D-Rendering of an *Amphistegina lessonii* specimen. An X-ray photograph of an *A*. *lessonii* specimen, the F-chamber is highlighted in yellow (Panel A). The F-chamber was virtually separated from the foraminifer to calculate the volume of the carbonate (Panel B).

**Fig 6:**
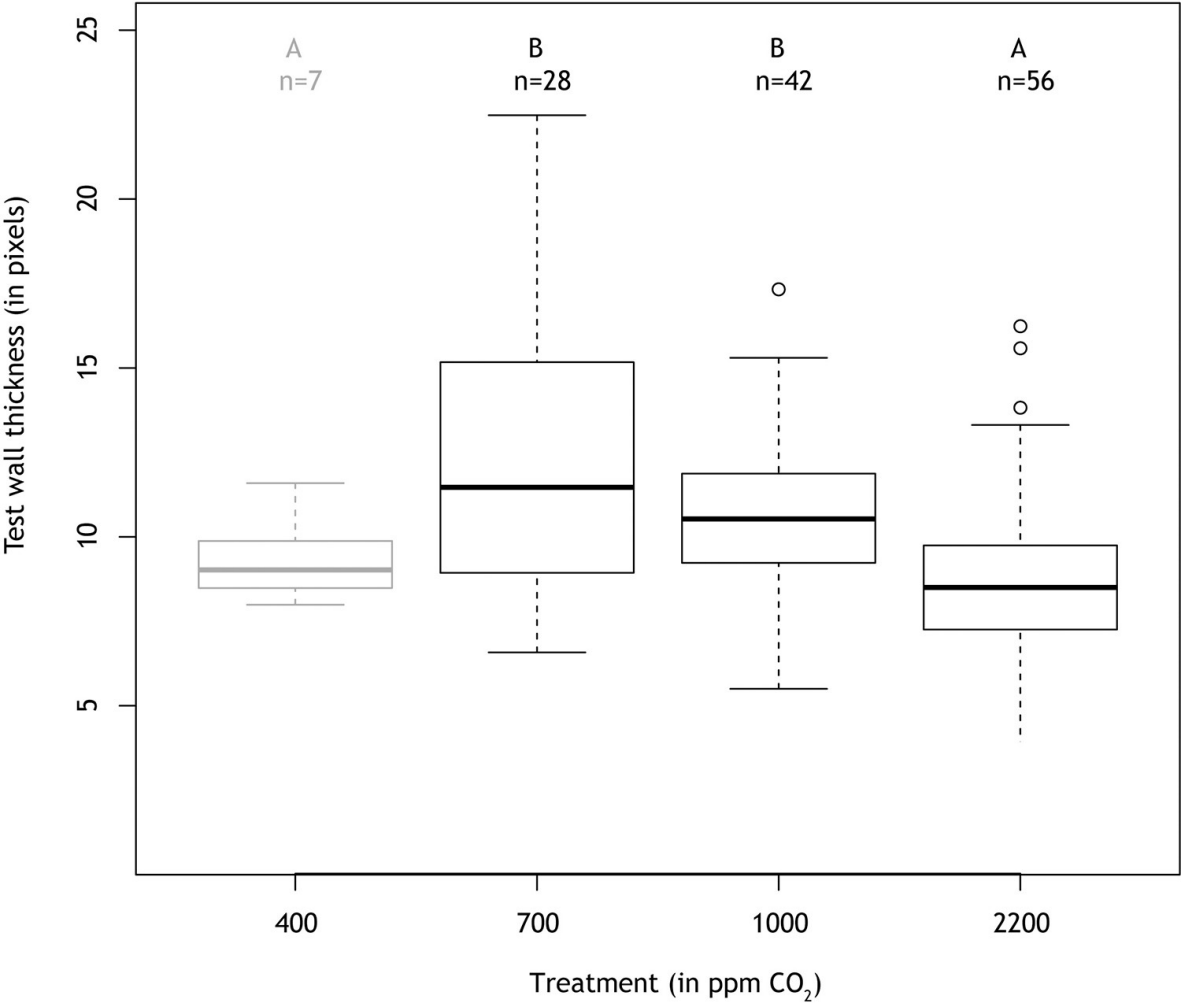
Dependence of test wall thickness on *p*CO_2_. The test wall thickness of F-chambers of *A*. *lessonii* specimens grown during the experiment varies with atmospheric *p*CO_2_ levels. Specimens exposed to moderately elevated CO_2_ concentrations show on average thicker shell walls than specimens grown under low as well as very high CO_2_ concentrations. Letters above the boxplots indicate statistically significantly different groups (t-test, p-value < 0.05). The 400 ppm treatment is shown in grey, due to the comparatively small number of thickness measurements used.

Similar to the number of chambers, the average test wall thickness, determined from MicroCT-Scans (Fig 5), in *A*. *lessonii* also varies with *p*CO_2_, with thickest walls at *p*CO_2_ levels of 700 and 1000 ppm (Fig 6). Based on isolation of the complete final chamber from the MicroCT-scan, the volume of calcite of the F-chamber from a single specimen from the 700 ppm treatment was determined to be close to 300,000 μm^3^, which equals approximately 8 nmol of CaCO_3_. In one specimen from the 2200 ppm treatment, the added calcite amounted to 47 nmol. The associated change in TA for this addition is lower than the total observed decrease in TA (S1 Table) by 2 and 10-fold at 2100 and 700 ppm, respectively.

## Discussion

### Calcification in foraminifera as a function of *p*CO_2_

A moderate increase in dissolved CO_2_ had a positive effect on calcification, both for *Amphistegina lessonii* and *Heterostegina depressa*. Most chambers were added at 700 ppm (Table 2) and for *A*. *lessonii*, most specimens (85%; Table 2) calcified at this *p*CO_2_. The total amount of carbonate produced, based on the changes in TA, was also highest for both species at 700 ppm *p*CO_2_ (Fig 3) and for *A*. *lessonii*, newly added chamber walls were thicker at 700 and 1000 ppm (Fig 6).

At the highest CO_2_ concentration (2200 ppm), both species added the least number of new chambers (Table 2) and the chamber walls of *A*. *lessonii* were thinnest at 400 and 2200 ppm (Fig 6). Our results show that determining foraminiferal calcification by counting new chambers, determining the chamber wall’s thicknesses and by monitoring the decrease in alkalinity, all reveal a similar response of calcification to *p*CO_2_. It may be that calcification does not scale with *p*CO_2_ linearly (or parabolically), but that the direction in the change of the saturation state also matters. Such hysteresis [56] is reported for coral reefs and is visible on daily changes in pH due to photosynthesis. Such a potential effect in foraminifera will be harder to find due to the intermittent nature of calcification in rotaliid foraminifera, but we cannot exclude the possibility that hysteresis may influence their chamber addition process.

The moderately positive influence of CO_2_ on calcification as observed here is in contrast with the response of the majority of marine calcifiers [57] and reports on the effect of CO_2_ on larger benthic foraminifera [58]. Although other studies have found mixed responses of large benthic foraminifera to elevated *p*CO_2_/reduced pH [17, 59] and showed that they are able to withstand periodic undersaturated conditions [60]. Finally, Fujita et al. [61] reported an optimum growth rate for three species of the large, high-Mg/Ca benthic foraminifera, similar to the pattern reported here. Although the number of incubated and analyzed specimens is smaller for *H*. *depressa* than *A*. *lessonii*, sensitivity to *p*CO_2_ of the latter appears strongest (Table 2). This is in line with a previous study reporting no changes in growth rates in *H*. *depressa* at *p*CO_2_ values between 467 and 1952 ppm [62], possibly indicating a strong decrease in calcification in this species for high (>2000 ppm) CO_2_ concentrations.

The (positive) response of large benthic foraminifera to elevated *p*CO_2_ likely involves the photosynthetic symbionts residing inside the shells of these foraminifera (diatoms for genera like *Amphistegina*, dinoflagellates for genera like *Amphisorus*; [63, 64]. The additional CO_2_ likely increases the delivery of nutrients and carbon from the symbionts to the foraminifera [65] and thereby provides an indirect link between carbon dioxide and calcification. Such a link would imply that symbiont density and surface-to-volume ratio of the foraminifer influence the effect of seawater CO_2_ concentration on calcification. Any negative impact (e.g., by increasing the energy costs associated with the raised intracellular-extracellular pH gradient) by the CO_2_-induced acidification may not outweigh the positive influence of enhanced photosynthetic activity. Furthermore, if these effects do not scale linearly with the concentration of CO_2_, pH or saturation state, their added effect may give rise to a net hyperbolic response of calcification to *p*CO_2_.

For smaller benthic and planktonic foraminiferal species, many studies indicated a negative response of calcification (either growth rate or size-normalized weight) even with a modest increase in *p*CO_2_ [29, 66–72]. These results may indicate that either calcification itself is affected by higher *p*CO_2_/lower saturation states or that other metabolic processes are hampered by elevated *p*CO_2_ and that calcification is indirectly affected. These studies, however, all focused on low-Mg/Ca species (e.g., planktonic foraminifera or *Ammonia* spp.).

The overall clear effect of elevated *p*CO_2_ on low-Mg benthic and planktonic foraminifera compared to the mixed, moderate, or even positive response by large benthic foraminifera may indicate that the mechanism responsible for incorporating Mg (or the lack thereof) is related to the pathway through which the inorganic carbon is taken up for calcification. This is in line with reports of inorganic carbon manipulation experiments on the incorporation of cations [29, 73] and was more recently provided with a mechanistic basis in which the inward calcium pumping is directly responsible for the uptake of inorganic carbon [36]. The coupling between inorganic carbon uptake and Mg partitioning would explain the much higher sensitivity of the low Mg/Ca planktonic foraminifera to seawater carbonate chemistry at relatively low *p*CO_2_ values [68]. Alternatively, the distinction between these foraminifera in their response to elevated *p*CO_2_ may be a function of their ecology and/ or presence of photosynthetic symbionts.

### Chamber addition versus calcification

At intermediate *p*CO_2_ values (700 ppm), the two foraminifera species investigated here produce most CaCO_3_. Although chamber addition rate (Fig 2) has been hypothesized to primarily reflect growth of the protoplasm which resides in the shell [74], we here observe a good correlation with shell wall thickness and alkalinity change. Protoplasmic growth likely follows food uptake, but here we also find a relation with carbonate chemistry of the water. This suggests that the cost of calcification is affecting the overall well-being of the foraminifera, potentially through the energetic cost of calcification especially at low pH [12, 75]. When a foraminifer is calcifying relatively well (i.e., produces a thick chamber wall) the reduced energetic costs may result in increased growth and hence also chamber addition. This may imply that chamber addition itself is hence indirectly coupled to calcification rate.

When comparing the precipitated calcite as calculated from the reduction in alkalinity and the number of chambers added, each chamber would consist of 10 to 15 micrograms of calcite (Fig 4). This is much more than the approximately 1 microgram of carbon estimated by the weight of individual tests divided by the average number of chambers making up the shell (average weight of adult *Amphistegina* [54]). As the last chambers in rotaliid species are larger and potentially contain relatively much calcite, we also calculated the volume of the carbonate added for two selected MicroCT-scanned specimens. This confirmed the offset between alkalinity based total amount of carbonate precipitated and the estimated carbonate based on chamber addition. The offset appears too large to be explained with factors such as the carbonate added on the older parts of the shell in addition to the added chamber itself (i.e., by lamellar calcification; [76]). The fact that some of the specimens were heavier calcified than others does not explain the observed offset either. Still, the evident correlation between the alkalinity and shell thickness also suggests a common response to the imposed carbonate chemistry. This suggests that either inorganically within the culture flask, or biomediated through micro-organismal calcification, the overall calcification in the culture flasks supplemented the foraminiferal calcification rate changes.

### Calcification in a future ocean

A calcification optimum of large benthic foraminifera at increased *p*CO_2_ levels would result in increased calcification with rising anthropogenic atmospheric CO_2_ concentrations within the next decades, which in turn would lead to a weak, but significant, positive feedback loop with CO_2_ concentrations [77], since calcification is a net source for dissolved CO_2_ [78]. Due to the interplay of acidification, warming and pollution it is difficult to assess precisely how larger benthic foraminiferal diversity and abundances will develop in the near future. If indeed elevated dissolved CO_2_ is beneficial for foraminiferal calcification, their deterioration due to climate change and pollution may be partly mitigated and adds to the uncertainty in prediction the contribution of large benthic foraminifera on reef stability, sediment production and shallow reef biogeochemical cycling [79]. With rates as high as 1 kg/m^2^/yr [15, 80, 81], larger benthic foraminifera can contribute severely to these processes locally.

Ries et al. (2009) suggest that these major differences could be related to a large range of factors such as species’ ability to regulate the pH at their site of calcification, whether a species’ skeleton is covered by an organic lining that separates the carbonate from direct contact to the sea water, or whether or not photosynthetic symbionts are involved. Especially the ability for pH regulation plays a major role for many calcifiers [12], and foraminifera are known to strongly manipulate the pH at their site of calcification and microenvironment [36, 82]. For *Amphistegina lobifera* a strong decrease in their microenvironmental pH caused by respiration and photosynthesis has been observed, with stronger decreases under higher atm *p*CO _2_ levels [27]. *Ammonia* sp. actively pumps out protons during calcification [36], resulting in a strong decrease in the pH of the sea water in the direct microenvironment of the shell, leading to shift in the speciation of inorganic carbon. At the lowered pH relatively more inorganic carbon occurs in the form of CO_2_ instead of bicarbonate, which allows diffusion through the foraminiferal membrane into the cell, due to the strong gradient in concentration. Pumping out protons not only caused a decrease of pH in the microenvironment, but also a corresponding increase of the pH within the cell [8], adding to the concentration gradient between sea water and the site of calcification. At the higher internal pH, the inorganic carbon speciation shifts again, causing CO_2_ to become CO_3_^2-^, which is then used by the cell to form CaCO_3_ [36].

Whether this mechanism is present in all benthic foraminifera, including *A*. *lessonii* and *H*. *depressa*, remains to be investigated. Differences in the chemical composition of the shells of genera like *Ammonia*, *Amphistegina* and *Heterostegina* [39, 83] suggest that they differ in the way they acquire the ions necessary for calcification [30, 84]. Finding the exact mechanisms by which they differ in their biomineralization pathway may also shed light on the difference in their response to elevated *p*CO_2_. With substantial differences amongst foraminifera in their response to increased *p*CO_2_, future changes in the balance between groups may be far more important for the marine inorganic carbon cycling than the current net foraminiferal calcification response to ocean acidification.

## Conclusion

Growth and calcification responses of large, benthic, symbiont bearing foraminifera *Heterostegina depressa* and *Amphistegina lessonii* to ocean acidification were tested by exposing them to 400, 700, 1000 and 2200 ppm of *p*CO_2_. Calcification by these foraminifera was measured directly using the Alkalinity Anomaly Technique, counting new chambers formed and using MicroCT-scans to analyze test properties such as thickness and carbonate volume for *A*. *lessonii*. Carbonate production by *A*. *lessonii* shows an optimum at slightly elevated *p*CO_2_ levels (700 ppm), but a decrease when *p*CO_2_ levels increased further. Chamber addition rates as well as carbonate production rates by *H*. *depressa* follow a similar pattern.

Benthic foraminifera are thus not only able to maintain calcification in moderately acidified conditions, but some species might even calcify significantly more at elevated *p*CO_2_ levels. Our results indicate that they profit from an increased amount of inorganic carbon present, before the negative effects of ocean acidification, such as higher energy costs to increase their internal pH sufficiently, tip the balance towards net negative effects.

## Supporting information

S1 Fig Relationship between CO_2_ and ΔTA. For both species the change in Total Alkalinity varies from week to week, but also between treatments. For *A*. *lessonii*, TA decreased more in the two lowest CO_2_ treatments, for *H*. *depressa* the strongest decrease in TA was observed at 700 ppm. The smallest change in TA for *A*. *lessonii* was observed at 1000 and 2200 ppm, for *H*. *depressa* at 2200 ppm. Mean values are indicated by plus symbols. Letters below the boxplots indicate statistically significantly different groups (t-test, p-value < 0.05). Note that the scale on the y-axis is reversed: a stronger decrease in TA indicates a more calcite produced during the experimental period.
(TIF)

S2 Fig Histogram of chambers added during the experiment per treatment for the pre-stained sub-set of specimens for A) *A. lessonii* and B) *H. depressa*. The duration of the experiment was 50 days for *A. lessonii* and 36 days for *H. depressa*. Since pre-staining with calcein did not work for the *H. depressa* specimens in the 1000 ppm treatment, no chamber counts could be determined. While peaks for all treatments were around 2–3 chambers for *H. depressa*, more specimens build a higher number of chambers in the 700ppm atm pCO_2_ treatment than during both higher and lower CO_2_ levels. A very similar pattern can be observed for *A. lessonii*.
(TIF)

S1 Table Changes in TA for each experiment. Negative values indicate a decrease in TA over time and hence reflect a net CaCO_3_ production.
(DOCX)

S1 File Number of new chambers formed during the experiment by the pre-stained individuals per experiment. (CSV)
