# Supplementary figures and images for: Impact of dissolved CO2 on calcification in two large, benthic foraminiferal species
Source: PLoS One. 2023 Aug 16;18(8):e0289122. doi: 10.1371/journal.pone.0289122 (PMC10431644; doi:10.1371/journal.pone.0289122)

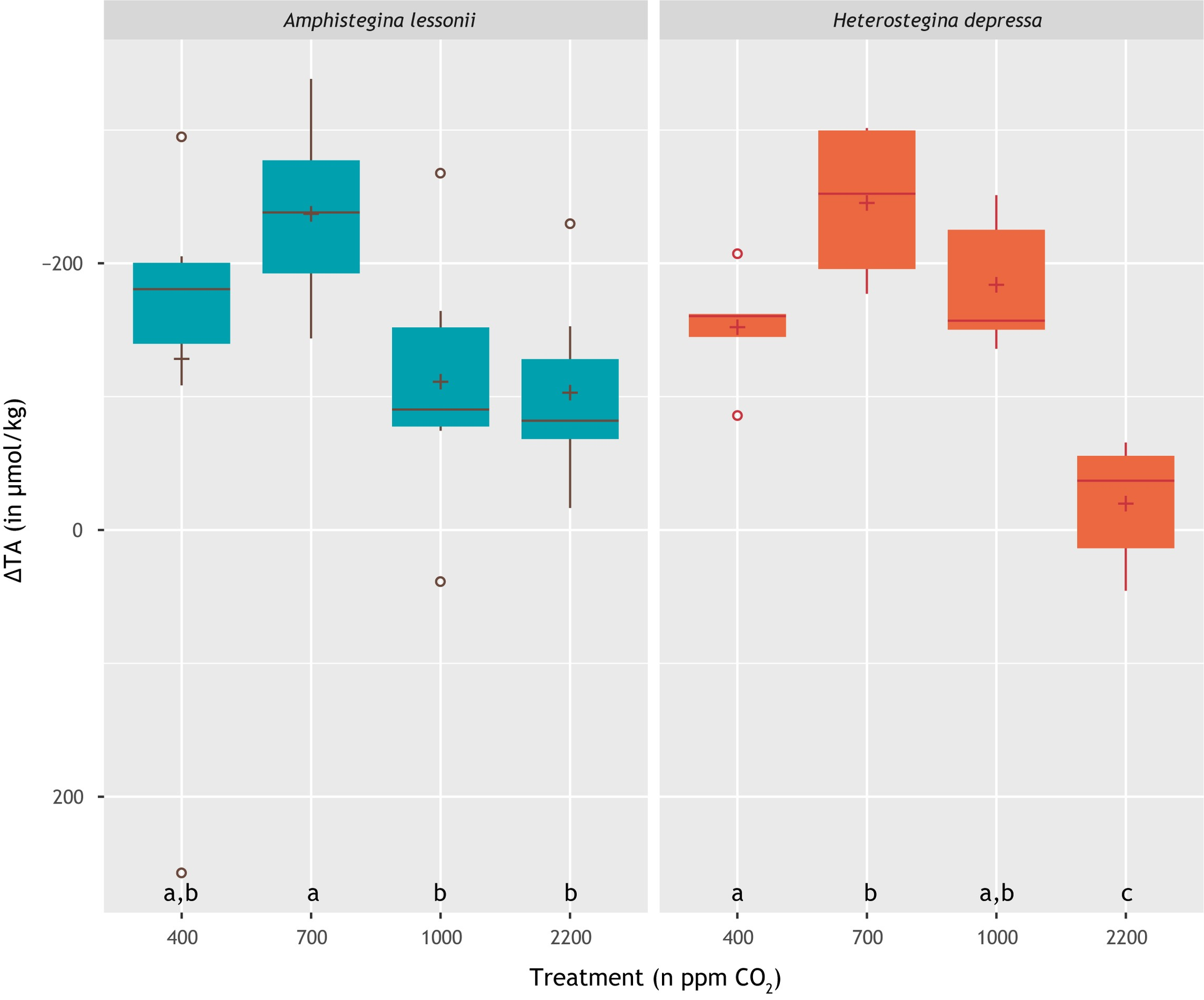

Supplement: S1 Fig — For both species the change in Total Alkalinity varies from week to week, but also between treatments. For A. lessonii, TA decreased more in the two lowest CO2 treatments, for H. depressa the strongest decrease in TA was observed at 700 ppm. The smallest change in TA for A. lessonii was observed at 1000 and 2200 ppm, for H. depressa at 2200 ppm. Mean values are indicated by plus symbols. Letters below the boxplots indicate statistically significantly different groups (t-test, p-value < 0.05). Note that the scale on the y-axis is reversed: a stronger decrease in TA indicates a more calcite produced during the experimental period. (TIF) [file pone.0289122.s001.tif]

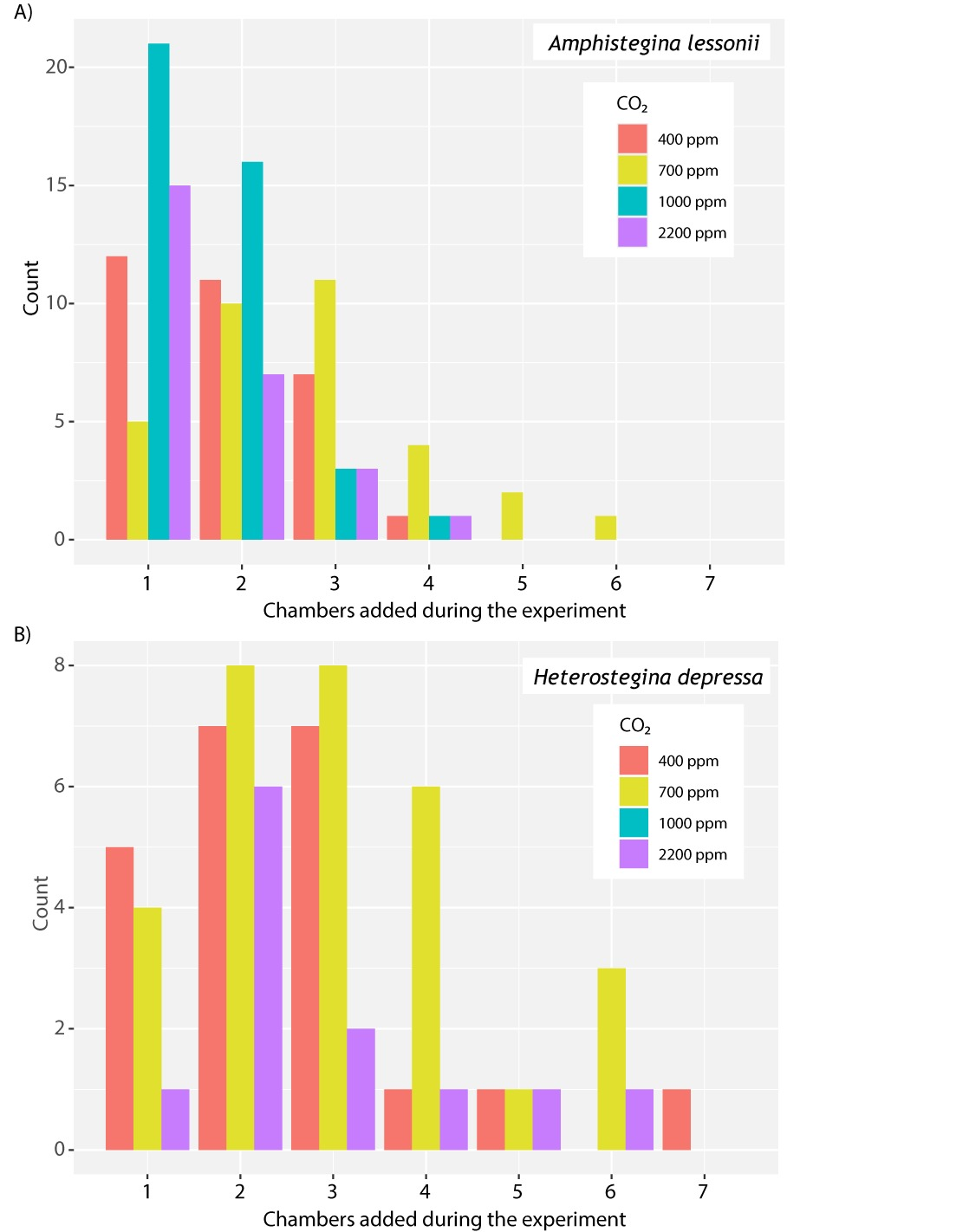

Supplement: S2 Fig — Histogram of chambers added during the experiment per treatment for the pre-stained sub-set of specimens for A) A. lessonii and B) H. depressa. The duration of the experiment was 50 days for A. lessonii and 36 days for H. depressa. Since pre-staining with calcein did not work for the H. depressa specimens in the 1000 ppm treatment, no chamber counts could be determined. While peaks for all treatments were around 2–3 chambers for H. depressa, more specimens build a higher number of chambers in the 700ppm atm pCO2 treatment than during both higher and lower CO2 levels. A very similar pattern can be observed for A. lessonii. (TIF) [file pone.0289122.s002.tif]
