# Supplementary material for: Impact of dissolved CO2 on calcification in two large, benthic foraminiferal species
Source: PLoS One. 2023 Aug 16;18(8):e0289122. doi: 10.1371/journal.pone.0289122 (PMC10431644; doi:10.1371/journal.pone.0289122)
Supplement: S1 Table — Negative values indicate a decrease in TA over time and hence reflect a net CaCO3 production. (DOCX) [file pone.0289122.s003.docx]

**Supporting Information**

**Table S1. Changes in TA for each experiment.** Negative values indicate a decrease in TA over time and hence reflect a net CaCO3 production.

|  | Average changes in TA per week  (μmol/kg) ± 1SD | |
| --- | --- | --- |
| *p*CO_2_ (ppmv) | *A. lessonii* | *H. depressa* |
| 400 | -193 ±60 | -152 ±44 |
| 700 | -237 ±66 | -245 ±58 |
| 1000 | -136 ±74 | -184 ±51 |
| 2200 | -103 ±69 | -53 ±14 |
